# Case report: Cytokine and miRNA profiling in multisystem inflammatory syndrome in children

**DOI:** 10.3389/fmed.2024.1422588

**Published:** 2024-08-01

**Authors:** Yun-Hao Tsai, Jun-Jie Hong, Chao-Min Cheng, Mei-Hsiu Cheng, Cheng-Han Chen, Min-Ling Hsieh, Kai-Sheng Hsieh, Ching-Fen Shen

**Affiliations:** ^1^School of Medicine, National Cheng Kung University, Tainan, Taiwan; ^2^Department of Taiwan Business Development, Inti Taiwan, Inc., Hsinchu, Taiwan; ^3^Institute of Biomedical Engineering, College of Engineering, National Tsing Hua University, Hsinchu, Taiwan; ^4^Department of Emergency Medicine, Taipei Veterans General Hospital, Taipei, Taiwan; ^5^School of Medicine, National Yang Ming Chiao Tung University, Taipei, Taiwan; ^6^Department of Pediatrics, National Cheng Kung University Hospital, College of Medicine, National Cheng-Kung University, Tainan, Taiwan; ^7^Department of Pediatrics and Structural, Congenital Heart and Echocardiography Center, School of Medicine, China Medical University, Taichung, Taiwan; ^8^Institute of Clinical Medicine, College of Medicine, National Cheng Kung University, Tainan, Taiwan

**Keywords:** MIS-C, COVID-19, cytokines, miRNA, IL-6, IL-1β, case report

## Abstract

Multisystem inflammatory syndrome in children (MIS-C) is an imperative pediatric inflammatory condition closely linked to COVID-19, which garners substantial attention since the onset of the pandemic. Like Kawasaki illness, this condition is characterized by an overactive immune response, leading to symptoms including pyrexia, cardiac and renal complications. To elucidate the pathogenesis of MIS-C and identify potential biomarkers, we conducted an extensive examination of specific cytokines (IL-6, IL-1β, IL-6R, IL-10, and TNF-α) and microRNA (miRNA) expression profiles at various intervals (ranging from 3 to 20 days) in the peripheral blood sample of a severely affected MIS-C patient. Our investigation revealed a gradual decline in circulating levels of IL-6, IL-1β, IL-10, and TNF-α following intravenous immune globulin (IVIG) therapy. Notably, IL-6 exhibited a significant reduction from 74.30 to 1.49 pg./mL, while IL-6R levels remained consistently stable throughout the disease course. Furthermore, we observed an inverse correlation between the expression of hsa-miR-596 and hsa-miR-224-5p and the aforementioned cytokines. Our findings underscore a robust association between blood cytokine and miRNA concentrations and the severity of MIS-C. These insights enhance our understanding of the genetic regulatory mechanisms implicated in MIS-C pathogenesis, offering potential avenues for early biomarker detection and therapy monitoring through miRNA analysis.

## Introduction

MIS-C represents a rare syndrome observed in pediatric patients following SARS-CoV-2 infection. Diagnosis hinges on specific criteria: patients under 21 years old presenting with fever, elevated levels of one or more inflammatory markers (such as C-reactive protein, erythrocyte sedimentation rate, fibrinogen, etc.), hospitalization, involvement of two or more organ systems (including cardiac, renal, respiratory, hematologic, gastrointestinal, dermatologic, or neurological), and confirmation of SARS-CoV-2 through RT-PCR, serology, antigen testing, or recent COVID-19 contact within 4 weeks before symptom onset (1). Predominantly affecting children aged 6–12, MIS-C commonly presents with gastrointestinal, cardiovascular, mucocutaneous, and respiratory symptoms (2).

While the precise pathophysiology of MIS-C necessitates further clinical exploration, many patients exhibit marked improvement following treatment with immune modulators (3). MIS-C pathogenesis involves dysregulation of the innate immune system, primarily accentuating the IL-1β pathway and elevating pro-inflammatory cytokine levels. Reports indicate heightened concentrations of IL-1β, IL-6, IL-8, IL-10, IL-17, and IFN-γ during the acute phase, with cytokine levels typically normalizing later (4).

Accordingly, treatment strategies predominantly aim to quell the excessive inflammatory response, drawing parallels from the management of conditions like Kawasaki disease. Intravenous immunoglobulin (IVIG) stands as the primary therapeutic intervention for MIS-C, with steroids reserved as a secondary option, usually for moderate to severe cases (5). Given IVIG’s anti-inflammatory properties and successful application in treating fulminant myocarditis associated with COVID-19 infections, it remains a cornerstone of MIS-C treatment (6).

MicroRNAs (miRNAs) play pivotal roles in mRNA silencing. Derived from DNA, they exert regulatory control over gene expression by binding to specific mRNA sequences, thereby influencing a significant array of human genes and biological functions. Recently, miRNAs have garnered attention as potential biomarkers for COVID-19.

In a study conducted by Mohamed et al. (7), the expression levels of serum miRNA-106a and miRNA-20a, along with inflammatory cytokines (TNF-α, IFN-γ, IL-10), and Toll-like receptor 4 (TLR4) were investigated in COVID-19 patients. Their investigation revealed an association between elevated TLR4 expression and downregulation of miRNA-20a with increased disease severity. Furthermore, a negative correlation was observed between miRNA-20a and TLR4 levels, suggesting potential therapeutic or predictive implications for these two biomarkers in COVID-19 management (7).

Overall, the pathogenesis of MIS-C is characterized by an aberrant immune response involving cytokines and microRNAs. Understanding the role of these molecules is crucial for unraveling the underlying mechanisms of the disease. This study focuses on elucidating the levels of cytokines and microRNA expression in a severely afflicted MIS-C patient undergoing IVIG treatment. The primary objective is to discern potential biomarkers while shedding light on the genetic regulatory pathways implicated in MIS-C pathogenesis.

## Case description

A 8-year-10-month-old girl was admitted from the Emergency Department with a primary complaint of intermittent fever reaching up to 40°C for 3 days on July 1, 2022 (D1). She was previously diagnosed with COVID-19 on June 8th, 2022. This time, she also had a minor cough with sputum, sore throat, a single episode of vomiting, and loose stools. Her dietary consumption has also been reduced. In the ED, she presented with a fever up to 39°C and her hemogram demonstrated white blood cell count (WBC) 9,900/ul, with differential counts of neutrophils (76.4%), lymphocytes (10.4%), monocytes (7.4%), eosinophils (3.1%), and basophils (2.7%). Additionally, her blood biochemistry testing revealed high hsCRP (6.52 mg/L), and elevated PCT (1.27 ng/mL).

After hospitalization, her condition worsened with the persistence of fever and the development of chest pain, and rapidly progressive into to cardiogenic shock, as indicated by hypotension and elevated cardiac enzyme levels, particularly Troponin-I (1.11 ng/mL). She was diagnosed with MIS-C based on the presence of fever, elevated CRP and PCT, cardiac and gastrointestinal involvement. In response to her deteriorating condition, she was transferred to the Pediatric Intensive Care Unit where she underwent treatment with IVIG totaling 51 grams (2 g/kg/dose) and dopamine continuous infusion.

Following this intervention, the patient’s condition gradually stabilized, and she was transferred to the general ward on July 12th, 2022 (Day 12). Throughout her hospitalization, we closely monitored various inflammatory markers, including D-dimer, ferritin, and hsCRP, which exhibited a gradual decrease. Meanwhile, the level of inflammatory cytokines (IL-6, IL-1β, IL-6R, IL-10, TNF-α) and miRNAs were measured later using ELISA assay and quantitative PCR, respectively. Importantly, she did not experience any further episodes of fever. Considering her stable condition and improving inflammatory markers, the decision was made to discharge the patient, with plans for outpatient follow-up care.

## Methods

### ELISA assay

The cytokine concentration in the patient’s blood was measured using ELISA. In the experiment, ELISA kits from R&D systems were used and the kit numbers of individual cytokines are D6050 for IL-6, DR600 for IL-6R, D1000B for IL-10, DLB50 for IL-1β, and DTA00D for TNF-α. The patient’s blood cytokine levels were quantified through ELISA. For this experiment, ELISA kits sourced from R&D Systems were employed, each corresponding to a specific cytokine. The kit identifiers for the individual cytokines were as follows: D6050 for IL-6, DR600 for IL-6R, D1000B for IL-10, DLB50 for IL-1β, and DTA00D for TNF-α.

### Total RNA extraction

Total RNA was isolated from 200 μL of plasma with miRNeasy Serum/Plasma Advanced Kit (Cat. no. 217204, Qiagen) following the manufacturer’s protocol. Plasma RNA sample was eluted in 20 μL nuclease-free water. The concentration of the extracted total RNA sample was quantified using Thermo Fisher’s Qubit microRNA Assay Kit (Q32880). A total of 200 μL of plasma was used to isolate the total RNA using the miRNeasy Serum/Plasma Advanced Kit (Cat. No. 217204, Qiagen) following the manufacturer’s instructions. The plasma RNA sample was then eluted in 20 uL of nuclease-free water. To determine the concentration of the extracted total RNA sample, the Thermo Fisher Qubit microRNA Assay Kit (Q32880) was employed.

### cDNA synthesis and qPCR analysis

miRNA candidate discovery services were provided by Inti Taiwan Inc. utilizing MIRAscan microRNA assay (Inti Taiwan Inc.). A total 2 ng of miRNAs from samples were used to synthesize cDNA in 20 μL reverse transcription reactions. The reverse transcription step was performed as follows: Poly-A tail was added to the RNA population using Poly-A polymerase, followed by cDNA synthesis with RNA Reverse Transcription Kit (Quark Biosciences, Inc.). Quantitative PCR was performed utilizing NextAmp™ Analysis System and MIRAscan PanelChip^®^ (designed by Inti Taiwan, Inc.; manufactured by Quark Biosciences, Inc.), which was pre-printed with miRNA specific primers. For qPCR analysis, 0.15 ng cDNA was added to the qPCR master mix (Quark Biosciences, Inc.), loaded into the MIRAscan PanelChip^®^, and qPCR were performed on Q Station™ (Quark Biosciences, Inc.) according to the following program: 95°C for 36 s and 60°C for 72 s for 40 cycles.

### Normalization of miRNA expression levels

The resulting miRNA expression profiles were normalized using the expression level of the qPCR spike-in control with the following formula:

delta Cq value = 20 – (Cq value – control). The higher the resulting value, the higher the miRNA expression level. miRNAs without amplification signals across all profiles were removed.

### Clustering analysis

The unsupervised hierarchical clustering analysis using R package heatmap demonstrated the correlation between samples and miRNA expression profiles. Based on the dendrogram, the expression profiles were divided into two groups.

### Correlation between miRNAs and cytokines

We used Pearson correlation to investigate the correlation between miRNA expression levels and cytokine concentrations. The Pearson correlation coefficient is a statistical measure of the strength and direction of a linear relationship between two variables. A positive coefficient indicates a positive association between the two variables, while a negative coefficient indicates a negative association.

### miRNA-target enrichment analysis

For miRNA enrichment analysis, we chose the miRNAs highly correlated with cytokine levels as input and performed microRNA target interaction (MTI) analysis using miRTarBase (8). miRTarBase is one of the largest experimentally validated databases for miRNA target analysis. We retained the MTIs with more than 1 strong evidence publication support or more than 2 weaker evidence or publications. Finally, the filtered target gene list was used for enrichment analysis using the R package clusterprofiler (9) according to the Kyoto Encyclopedia of Genes and Genomes (KEGG) Pathway (10).

## Results

### Changes in cytokine concentration levels over time

**Figure 1:**
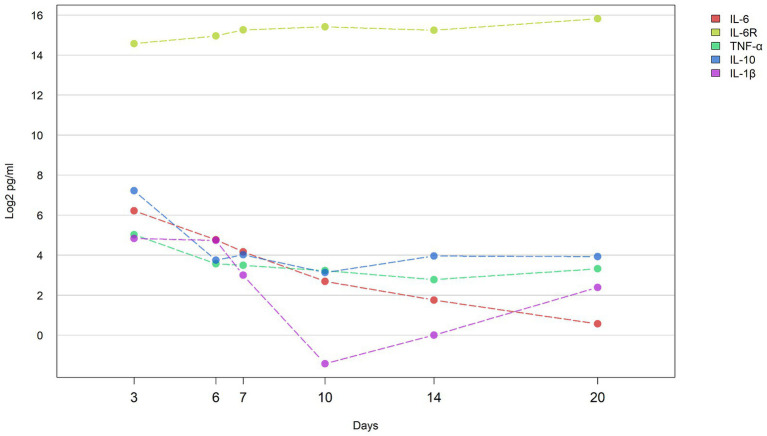
Concentrations of cytokines during hospital course the line graph shows the concentration of cytokines (IL-6, IL-1β, IL-6R, IL-10, and TNF-α) in the blood of patients at different time points (D3: 3 days, D6: 6 days, D7: 7 days, D10: 10 days, D14: 14 days, and D20: 20 days) change trend. Y axis represents log_2_ transformed cytokine concentration (pg/ml).

Serum levels of IL-6, IL-6R, TNF-α, IL-10, and IL-1β were assessed at six distinct time points (Days 3, 6, 7, 10, 14, Day 20) in the studied patient (Supplementary Table S1). Except for IL-6R, which exhibited a progressive increase in concentration from Days 3 to 20, the levels of the remaining four cytokines demonstrated an overall downward trend, with IL-6 displaying the most pronounced decrease (Figure 1). Specifically, the concentration changes for each cytokine were as follows: IL-6R increased from 24320.6 to 57531.6 pg./mL, IL-6 decreased from 74.3 to 1.5 pg./mL, IL-1β decreased from 28.5 to 5.2 pg./mL, IL-10 decreased from 148.5 to 15.2 pg./mL, and TNF-α decreased from 32.6 to 10.0 pg./mL.

### miRNA expression levels over time by hierarchical clustering

**Figure 2:**
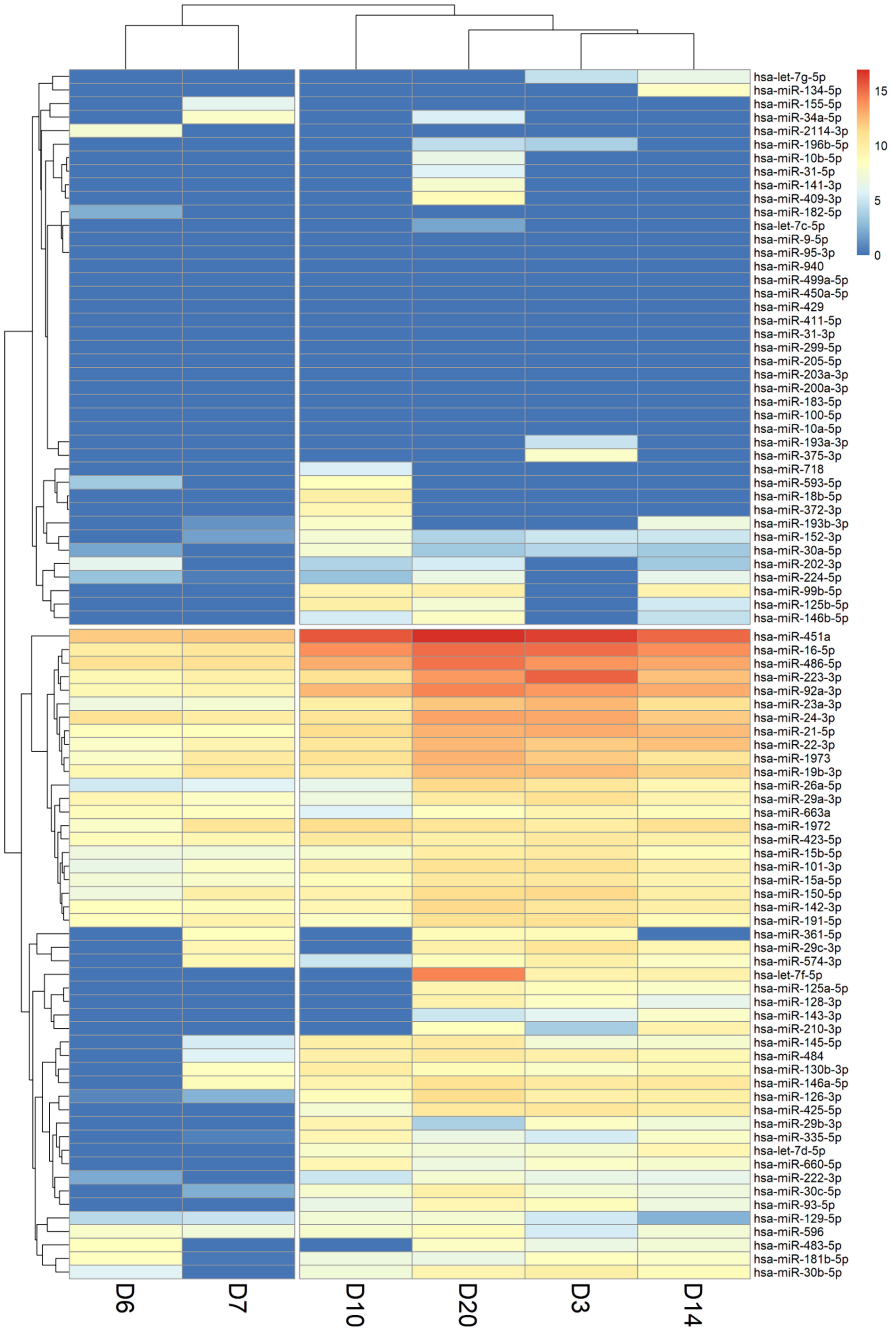
Heatmap of miRNA expression levels in patient blood samples at six-time points (D3: 3 days, D6: 6 days, D7: 7 days, D10: 10 days, D14: 14 days, and D20: 20 days). A value of 0 indicates that the miRNA was not expressed or not detected (shown in dark blue). The X-axis shows the order after the hierarchical cluster analysis of the six-time point samples, and the Y-axis shows the order after the hierarchical cluster analysis of miRNAs.

Hierarchical clustering was utilized to classify and categorize miRNA samples based on their similarities or dissimilarities, creating a hierarchical structure (Figure 2). We observed a consistent lack of expression in the miRNAs located in the upper section of the heatmap across various time points. This lack of expression may be potentially attributed to constraints encountered during the sample collection process. miRNAs that consistently showed no expression at all time points were subsequently excluded from further analyses. From the X-axis of the heat map, the relationships between samples were divided into two large categories based on the branches of dendrogram. It was observed that the miRNA expression levels of the two samples on the left (D6 and D7) were highly consistent, and this consistency coincided with the time point when the cytokine levels in the early stages of patient treatment started to gradually decrease. In addition, four-time points on the right (D3, D10, D14, D20) were classified into another category. At the D10 time point, many cytokines had already returned to baseline levels. Cytokine levels in the blood of patients at the D14 and D20 time points had also returned to normal levels while the patient was recovering in the general ward. Interestingly, the D3 time point was also included into this category, despite being the period with the highest inflammatory index in pediatric patient. This could be attributed to the influence of miRNAs that did not show expression in the clustering results.

### miRNA and cytokine correlation

**Figure 3:**
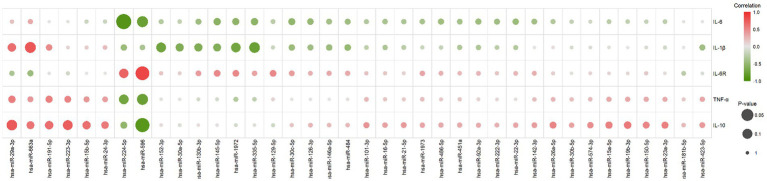
The correlation between changes in cytokine concentrations and miRNA expression levels. It was calculated using the Pearson correlation, with positive scores indicating a positive correlation between both (marked with a gradient of red) and negative scores indicating a negative correlation between both (marked with a gradient of green). The size of the circles in the graph represents the significance of the correlation test, larger circles indicate higher significance.

Pearson correlation was employed to calculate the correlation between results obtained from the experiments for cytokine and miRNA detection. A positive correlation coefficient (*r*-value) signifies a positive correlation between the changes in cytokine concentration levels and miRNA expression level changes, whereas a negative correlation coefficient indicates a negative correlation between the two (Figure 3). Cytokine-miRNA pairs with a correlation coefficient exceeding 0.7 were identified, including IL-6R and hsa-miR-224-5p (*r* = 0.70), IL-6R and hsa-miR-596 (*r* = 0.86), IL-10 and hsa-miR-223-3p (*r* = 0.72), IL-10 and hsa-miR-29a-3p (*r* = 0.75), and IL-1β and hsa-miR-663a (*r* = 0.77) (Supplementary Figure S1A). Cytokine-miRNA pairs with a correlation coefficient less than 0.7 were also identified, including IL-6 and hsa-miR-224-5p (*r* = −0.89), IL-6 and hsa-miR-596 (*r* = −0.77), TNF-α and hsa-miR-224-5p (*r* = −0.71), TNF-α and hsa-miR-596 (*r* = −0. 78), IL-10 and hsa-miR-596 (*r* = −0.87), IL-1β and hsa-miR-152-3p (*r* = −0.72), IL-1β and hsa-miR-1972 (*r* = −0.71), and IL-1β and hsa-miR-335-5p (*r* = −0.76) (Supplementary Figure S1B). This suggests that the changes in cytokine levels in MIS-C patients may be regulated by miRNAs. In addition, we also observed a correlation between the changes in IL-6 concentration at different time points and the changes in hsCRP concentration (*r* = 0.83) (Supplementary Figure S1C).

### miRNA functional analysis

**Figure 4:**
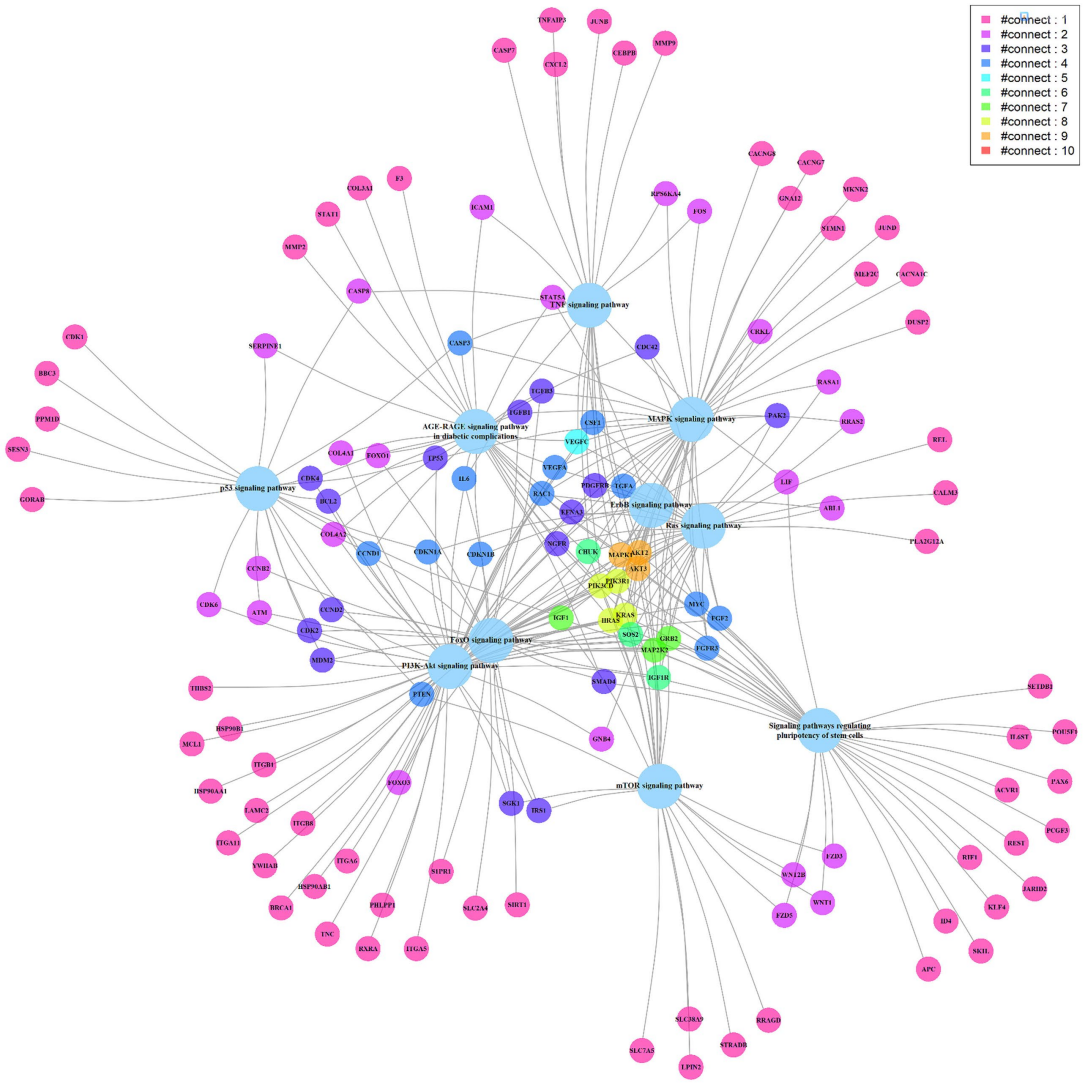
The miRNA targeted signaling pathway and genes network diagram. The big light blue circles represent signaling pathways, while the small circles represent genes. The edges connect the genes with the pathways, and the genes are depicted in different colors to represent the number of edges connecting them, with more edges indicating their involvement in multiple pathways.

To further understand the miRNA regulatory network, 8 miRNAs highly correlated with cytokine concentration changes were searched for related target genes in the miRTarBase database (Supplementary Table S2). These miRNA-regulated genes were then analyzed using the clusterProfiler tool for enrichment analysis and relevant signaling pathways were filtered out (Supplementary Table S3). The most statistically significant pathway was the PI3K-Akt signaling pathway, which was reported to be associated with the induction and generation of multiple cytokines. The pathway includes 58 genes regulated by miRNAs (Supplementary Figure S2) (11–13). Other pathways related to promoting cytokines include the MAPK signaling pathway, TNF signaling pathway, Ras signaling pathway, etc. (12, 14–17). We present the top 10 signaling pathways and their miRNA-regulated genes in a network diagram based on statistical significance (Figure 4). Genes participating in multiple signaling pathway functions are connected through various edges. Among them, MAPK1, AKT2, and AKT3 (marked in orange) participate in 9 signaling pathways. MAPK1 is a target gene of hsa-miR-335-5p, and AKT2 and AKT3 are target genes of hsa-miR-29a-3p (18–20)

## Discussion

### Relationship between MIS-C and cytokines

MIS-C is characterized by a transient upregulation of proinflammatory cytokines, notably interleukins and interferon gamma (IFN-γ) (21). Diagnosis of MIS-C heavily relies on elevated levels of interleukin-6 (IL-6), a key protein associated with immune response and inflammation (22–24).

Our research reveals a gradual decline in IL-6 levels following IVIG treatment, returning to baseline (3.38 pg./mL) by Day 14, while IL-6R levels exhibit an upward trend. This suggests a resolution of the inflammatory state and increased resilience to subsequent inflammatory challenges. IL-6, IL-6R, IL-1β, IL-10, and TNF-α play intricate roles in MIS-C, underscoring the importance of further exploration into cytokine involvement in MIS-C (25).

### Relationship between MIS-C and microRNAs

In our investigation, we examined the expression profiles of various miRNAs in patients with MIS-C. Notably, hsa-miR-224-5p, hsa-miR-596, and hsa-miR-663a exhibited significant and promising alterations. These miRNAs play pivotal roles in cytokine regulation, suggesting their potential as novel biomarkers for monitoring disease progression.

### Key microRNAs and their associations with specific cytokines

#### Hsa-miR-224-5p

Previous studies indicated that hsa-miR-224 may modulate the inflammatory response by targeting PTX3 mRNA, a gene involved in the activation of the p65/NF-κB pathway (26). Additionally, downregulation of hsa-miR-224-5p has been observed in severe COVID-19 cases (27, 28). In our study, we identified a negative correlation between hsa-miR-224-5p and the concentrations of IL-6, TNF-α, IL-10, and IL-1β (correlation coefficients: −0.89, −0.71, −0.53, and − 0.46, respectively), suggesting a potential regulatory role in suppressing the inflammatory response in MIS-C patients.

#### Hsa-miR-596

COVID-19 is characterized by an exaggerated inflammatory response mediated by the mitogen-activated protein kinase (MAPK) pathway (29). Hsa-miR-596 targets MEK1 and apoptosis-related proteins, inhibiting MAPK/ERK signaling (30, 31). Our study revealed a significant negative correlation between hsa-miR-596 and the concentrations of IL-6 (*r* = −0.77), IL-10, TNF-α, and IL-1β (correlation coefficients: −0.87, −0.78, and-0.37, respectively), indicating its potential as a predictive biomarker for MIS-C severity.

#### Hsa-miR-663a

Computational analyses of hsa-miR-663 target genes have identified a binding site in the 3’UTR of the APC gene, a constituent of the Wnt signaling pathway (32). Previous investigations have underscored a reciprocal relationship between NF-κB signaling and the Wnt pathway, primarily characterized as a negative regulatory interaction (33, 34).

In our study, we observed a positive correlation between hsa-miR-663a expression levels and changes in IL-6, TNF-α, IL-10, and IL-1β concentrations (correlation coefficients: 0.19, 0.56, 0.75, and 0.64, respectively). This suggests a potential mechanism by which hsa-miR-663a promotes NF-κB signaling pathway activation by inhibiting the Wnt signaling pathway. Although direct evidence of hsa-miR-663a concentration alterations in MIS-C is lacking, research has proposed its upregulation as a potential predictive biomarker for SARS-CoV-2 infection (35).

## Conclusion

MIS-C involves dysregulation of the innate immune system post-infection, activating the IL-1β pathway and increasing cytokine levels. The novelty of this study lies in linking miRNA activity to cytokine responses, offering new insights into MIS-C pathogenesis. Our findings suggest that IVIG treatment regulates cytokine levels potentially mediated by miRNAs, highlighting miRNAs’ role in IVIG treatment mechanisms for MIS-C. This regulatory interplay between miRNAs and cytokines warrants further investigation, with potential implications for MIS-C pathogenesis, identification, and treatment monitoring. Identifying specific cytokines and miRNAs as biomarkers is crucial for future treatment advancements.

## Data availability statement

The original contributions presented in the study are included in the article/Supplementary material, further inquiries can be directed to the corresponding authors.

## Ethics statement

The studies involving humans were approved by the Institutional Review Board of China Medical University Hospital reviewed and approved the study protocol (IRB no. CMUH111-REC1-185). The studies were conducted in accordance with the local legislation and institutional requirements. Written informed consent was obtained from the minor(s)’ legal guardian/next of kin for the publication of any potentially identifiable images or data included in this article.

## Author contributions

Y-HT: Conceptualization, Writing – original draft. J-JH: Conceptualization, Writing – original draft. C-MC: Conceptualization, Writing – review & editing. M-HC: Data curation, Writing – original draft. C-HC: Data curation, Writing – original draft. M-LH: Data curation, Writing – review & editing. K-SH: Methodology, Writing – review & editing. C-FS: Supervision, Writing – original draft, Writing – review & editing.
