# Supplementary figures and images for: Case report: Cytokine and miRNA profiling in multisystem inflammatory syndrome in children
Source: Front Med (Lausanne). 2024 Aug 1;11:1422588. doi: 10.3389/fmed.2024.1422588 (PMC11324540; doi:10.3389/fmed.2024.1422588)

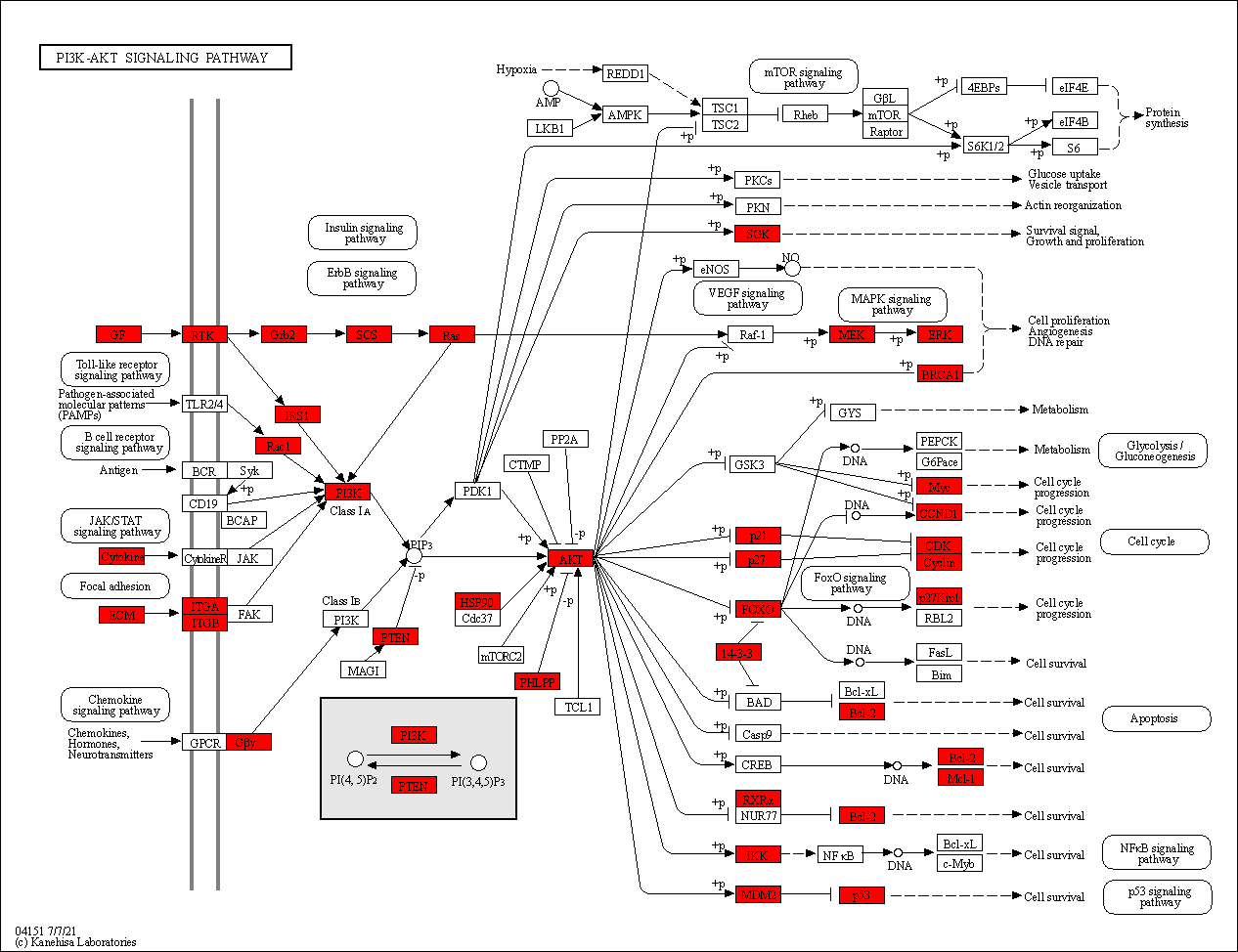

Supplement: Supplementary file 5 [file Image_2.PNG]
